# Outbreak of *Neisseria meningitidis* Conjunctivitis in Military Trainees — Texas, February–May 2025

**DOI:** 10.15585/mmwr.mm7433a1

**Published:** 2025-09-04

**Authors:** Susan J. Ching, Ga On Jung, Angela Osuna, Theresa Casey, Hui Xia, Karina Bostwick, Amol H. Patadia, Lauren M. Sweet, Oscar Gallardo-Huizar, Thomas F. Gibbons, Joseph E. Marcus

**Affiliations:** ^1^Trainee Health Surveillance, 559 THLS, Joint Base San Antonio-Lackland, Texas; ^2^Clinical Investigations and Research Support, Joint Base San Antonio-Lackland, Texas; ^3^Ophthalmology Service, Joint Base San Antonio-Lackland, Texas; ^4^Department of Surgery, Uniformed Services University of Health Sciences, Bethesda, Maryland; ^5^Infectious Disease Service, Brooke Army Medical Center, Joint Base San Antonio, Texas; ^6^Department of Medicine, Uniformed Services University of Health Sciences, Bethesda, Maryland.

**Summary**

**What is already known about this topic?**

Bacterial conjunctivitis is uncommon in immunocompetent adults. *Neisseria meningitidis* is an unusual bacterial cause of conjunctivitis.

**What is added by this report?**

Forty-one cases of *N. meningitidis* conjunctivitis caused by an unencapsulated (nongroupable) strain identified by whole genome sequencing occurred in young, healthy military trainees living in a communal setting; all had received quadrivalent meningococcal vaccine. No source was identified. One patient developed periorbital cellulitis and received intravenous antibiotics; all other patients were treated successfully with topical antibiotics.

**What are the implications for public health practice?**

When outbreaks of mucopurulent conjunctivitis occur in congregate living settings, culturing exudate can identify etiology, and whole genome sequencing can help guide treatment and outbreak response. Nongroupable *N. meningitidis* conjunctivitis in otherwise healthy persons might be successfully treated with topical antimicrobials.

## Abstract

Viral and allergic conjunctivitis are more common than bacterial conjunctivitis in healthy immunocompetent adults. *Neisseria meningitidis* is an uncommon cause of bacterial conjunctivitis. During February–May 2025, an outbreak of 41 meningococcal conjunctivitis cases occurred among healthy, communally housed, military trainees at Joint Base San Antonio-Lackland in San Antonio, Texas; all had received the quadrivalent meningococcal vaccine. One patient was hospitalized with periorbital cellulitis and received intravenous antibiotics; all other patients were treated successfully with topical antibiotics. Whole genome sequencing of isolates from the first two cases suggested that the organism was unencapsulated (nongroupable) and that the cases were related. After the identification of two cases of *N. meningitidis* conjunctivitis among military trainees within a 3-week period in February 2025, an investigation was initiated by the base health surveillance team. Investigation of basic trainee hygiene and cleaning practices found that all protocols were followed; no source for the outbreak was found. When outbreaks of mucopurulent conjunctivitis occur in congregate living settings, culturing exudate can identify outbreak etiology, and whole genome sequencing can help guide treatment and response. Previous studies indicated that systemic antimicrobial therapy might be needed to prevent invasive infections of *N. meningitidis* cases; findings from this investigation suggest that nongroupable *N. meningitidis* conjunctivitis in otherwise healthy persons might be successfully treated with topical antimicrobials.

## Introduction

In February 2025, two cases of *Neisseria meningitidis* bacterial conjunctivitis were identified in otherwise healthy basic military trainees at Joint Base San Antonio-Lackland in San Antonio, Texas; an investigation was conducted to identify the source of the outbreak and to make recommendations for treatment. Ultimately, 41 cases of *N. meningitidis* conjunctivitis and 32 cases of *Haemophilus* species conjunctivitis were identified among 11,797 trainees. This report describes the investigation and outbreak response, including the characteristics of the cases and the isolates, and response to treatment with topical antibiotics.

## Investigation and Results

### Housing and Prophylaxis for Trainees on Arrival at Joint Base San Antonio-Lackland

U.S. Air Force basic training takes place at Joint Base San Antonio-Lackland in San Antonio, Texas. Trainees are organized into rolling military units averaging 900 entering and graduating trainees per week, with each unit further divided into groups of approximately 52 trainees. Trainees are assigned to specific dormitories, which typically house 50–60 persons, sleeping in beds that alternate head directions, creating a foot-to-head orientation in the bay. Trainees arrive weekly and undergo a standardized 7.5-week basic military training (BMT) curriculum organized by week of training, resulting in training activities and associated exposures that are typically consistent for each class.

To prevent invasive meningococcal and streptococcal disease outbreaks, all trainees receive quadrivalent meningococcal (Groups A, C, Y, and W) (Menveo) vaccine within 72 hours of arrival and a single dose of penicillin G benzathine injectable suspension, respectively, within 7 days of arrival (*1*). Penicillin-allergic trainees receive weekly oral azithromycin to prevent streptococcal disease during BMT.

### Identification of First Two Conjunctivitis Cases

On February 5, 2025, a case of *N. meningitidis* bacterial conjunctivitis was identified in an otherwise healthy BMT trainee who had experienced 2 days of mucopurulent ocular discharge; the trainee had no known exposure to *N. meningitidis*. Although the symptoms initially suggested viral conjunctivitis (*2*), the copious unilateral discharge led the health care provider to culture the exudate, which was positive for *N. meningitidis* 6 days later. Microbial isolates were tested and identified by Vitek 2 (bioMerieux). On February 21, a second trainee (from a different training unit) was also evaluated for unilateral copious mucopurulent ocular discharge associated with periocular edema without corneal involvement. The culture of the exudate from this trainee’s eye was positive for *N. meningitidis*, raising concern about potential cases of invasive meningococcal disease. An investigation was initiated to identify additional cases, determine the risk for invasive meningococcal disease, and describe the patients and evaluate their response to treatment. The local human research protection program, Defense Health Agency San Antonio Market Office of Research Protocol Support, determined these activities not to be research.*

### Surveillance for Conjunctivitis

The first two cases, which occurred among trainees who started training 2 weeks apart, both occurred during both patients’ fourth week of BMT. On February 23, the day that the second patient’s culture result was received, the Trainee Health Surveillance team (a group of epidemiologists and preventive medicine physicians responsible for active and passive disease surveillance of the BMT population) established a registry and began active surveillance to identify cases of mucopurulent conjunctivitis and ensure that a sample was obtained for culture for each case. A confirmed case was defined as a positive *N. meningitidis* culture result from ocular discharge collected from a person with conjunctivitis. A probable case was defined as symptomatic conjunctivitis with exudate in a patient who had contact with a person with a confirmed case of meningococcal conjunctivitis but without laboratory confirmation. A suspected case was defined as symptomatic conjunctivitis with exudate in a person with no known contact with a confirmed case. Clinicians who worked in emergency departments or primary care on the base were requested to assist in active surveillance by submitting samples of ocular discharge from patients with conjunctivitis to the microbiology laboratory for culture and reporting suspected or probable cases to the Trainee Health Surveillance registry rather than providing standard empiric treatment without culture. Patients with confirmed meningococcal conjunctivitis received topical ocular erythromycin, ciprofloxacin, or moxifloxacin and were referred for an ophthalmologic assessment of corneal involvement.

### Identification of Additional Cases

**FIGURE 1:**
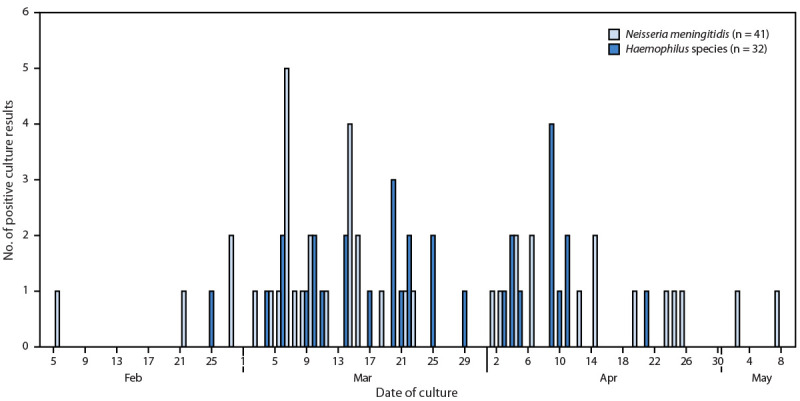
Number of positive bacterial ocular discharge culture results among military trainees, by date of culture and pathogen (N = 73)* — Joint Base San Antonio-Lackland, Texas, February 5–May 9, 2025 * Includes one basic military trainee instructor with conjunctivitis who received a positive *N. meningitidis *culture result on March 15, 2025.

**FIGURE 2:**
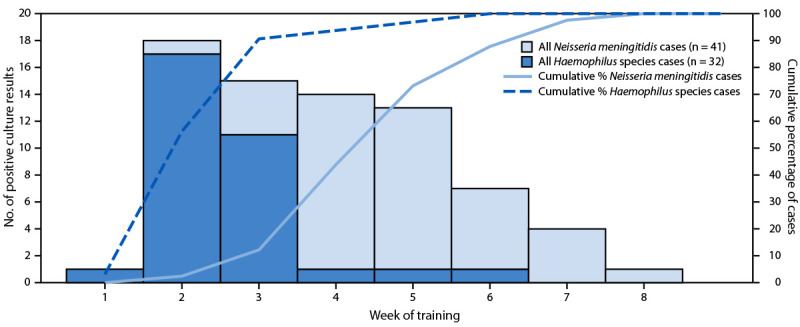
Number of positive bacterial ocular discharge culture results and cumulative percentage of positive results among military trainees, by week of training and pathogen (N = 73) — Joint Base San Antonio-Lackland, Texas, February–May 2025

During February 23–May 9, 2025, a total of 79 cases of mucopurulent conjunctivitis were identified among 11,797 trainees who started BMT in San Antonio (6.7 per 1,000); cultures from 41 (52%) patients were positive for *N. meningitidis*, and 32 (41%) were positive for *Haemophilus* species. Four (5%) patients received negative culture results, one patient’s ocular culture was positive for *Corynebacterium macginleyi*, a known cause of conjunctivitis (*3*), and for one patient, no specimen was collected for culture. Among the 41 laboratory-confirmed cases of *N. meningitidis* conjunctivitis, 23 (56%) occurred within 1 month of onset of the second case (Figure 1). Among the 32 *Haemophilus* species conjunctivitis cases, 29 (91%) were identified during the first 3 weeks of training, whereas 36 (87.8%) of the positive *N. meningitidis* ocular cultures were identified during or after the fourth week of training (Figure 2).

### Clinical Characteristics of Patients with *N. meningitidis* Conjunctivitis

The 41 confirmed cases of *N. meningitidis* conjunctivitis occurred in trainees in 37 unique BMT groups. During this period, men constituted 78% of the BMT population but accounted for 90% of the *N. meningitidis* cases. Among trainees with confirmed *N. meningitidis* conjunctivitis, 33 (80%) reported an antecedent upper respiratory infection (Table). Overall, 35 (85%) patients had unilateral eye involvement. All patients improved within 24 hours of starting treatment with topical moxifloxacin, ciprofloxacin, or erythromycin. One patient was hospitalized after a delay in initiating topical moxifloxacin that led to progression of infection to periorbital cellulitis, requiring a short course of intravenous antibiotics. No patients developed invasive corneal ulceration or orbital cellulitis. Contact tracing, including prophylactic antibiotics for close contacts, was deferred, because prophylaxis is currently recommended only for close contacts of persons with invasive disease (bacteremia or meningitis) (*4*). Whereas cases of *Haemophilus* species conjunctivitis occurred in patients who received either penicillin or azithromycin prophylaxis, *N. meningitidis* infections only occurred in patients who received penicillin.

**TABLE:** Characteristics of military trainees* with *Neisseria meningitidis* conjunctivitis cases (N = 41) — Joint Base San Antonio-Lackland, Texas, February–May 2025

Characteristic	No. (%)
**Average age, yrs (range)**	22 (17–39)
**Sex**	
Male	37 (90.2)
Female	4 (9.8)
**Week of training**	
1	0 (—)
2	1 (2.4)
3	4 (9.8)
4*	13 (31.7)
5	12 (29.3)
6	6 (14.6)
7	4 (9.8)
8	1 (2.4)
**Antecedent upper respiratory infection**	
Yes	33 (80.5)
No	8 (19.5)
**Signs or symptoms**	
Cough	27 (65.9)
Congestion	27 (65.9)
Sore throat	22 (53.7)
Rhinorrhea	15 (36.6)
Ocular pain	12 (29.3)
**Prophylactic antibiotics received at arrival**	
Azithromycin	0 (—)
Penicillin	40 (97.6)
None	1 (2.4)
**Wore eyeglasses**	
No	20 (48.8)
Yes	19 (46.3)
Unknown	2 (4.9)
**Involved eye**	
Right	19 (46.3)
Left	16 (39.0)
Both	6 (14.6)
**Hospitalized**	
No	40 (97.6)
Yes	1 (2.4)

* Includes one instructor with conjunctivitis and who received a positive *N. meningitidis* culture result on March 15, 2025, who was instructing a unit during the fourth week of training and did not receive penicillin or azithromycin prophylaxis.

### Whole Genome Sequencing

While serogrouping and sequencing are commonly performed by local, state, and federal public health laboratories for *N. meningitidis* isolates from cases of invasive meningococcal disease, neither is typically performed for isolates from noninvasive disease cases. However, after diagnosis of the second case, and to guide the public health response, whole genome sequencing of isolates from the first two ocular cultures was performed to determine whether they were related, predict antimicrobial resistance, and ascertain whether virulence factors associated with invasive disease were present. As has been reported for other cases of meningococcal conjunctivitis (*5*), both isolates were nongroupable, without the presence of c*saB*, *csb*, *csc*, *csw*, and *csy* genes associated with encapsulation, suggesting low risk for development of invasive disease. Sequencing demonstrated that the two isolates were both sequence type (ST) 32 and were closely related. ST-32 has previously been associated with meningococcal disease outbreaks caused by the encapsulated serogroup B *N. meningitidis*; however, because this strain was not encapsulated, it was not expected to cause invasive disease in otherwise healthy patients. The sequenced isolates indicated decreased susceptibility to penicillin based on a mutation in the *penA* gene, otherwise no other genetic correlates of antimicrobial resistance were identified.

### Environmental Investigation and Training Activity Evaluation

The Trainee Health Surveillance team evaluated dormitory cleanliness, including the showers and common areas, and reviewed established cleaning protocols. No environmental specimens were collected for testing. Various field training activities during the fourth week of training were also evaluated to ascertain their risk as a source of transmission and to confirm adherence to cleaning protocols. The health team who observed gas mask cleaning noted that staff members followed recommended cleaning and sanitizing protocols, using liquid sodium hypochlorite disinfecting solution (bleach) at recommended concentrations. As the outbreak continued, other potential common sources of transmission were investigated, including cardiopulmonary resuscitation training. However, because trainees did not practice rescue breathing on the mannequin, this activity posed a low risk for transmission. Evaluation of the military shooting range also did not identify any potential common source of transmission; safety goggles were not shared and were cleaned with hypochlorite disinfectant wipes at the end of each training session.

## Public Health Response

During February 13–18 (after identification of the first two cases), the Trainee Health Surveillance team conducted training site visits and provided a CDCinfographiconconjunctivitisprevention to the BMT staff members. Because the *N. meningitidis* conjunctivitis cases were noninvasive and caused by a nongroupable strain, postexposure prophylaxis was not recommended for close contacts of patients with confirmed cases and additional vaccination was not indicated (*4*).

## Discussion

Invasive meningococcal disease outbreaks have been associated with congregate living settings, including military or college dormitories; however, no reported outbreaks of meningococcal conjunctivitis were identified in the literature by an English-language search of Ovid MEDLINE via PubMed using keywords “primary meningococcal conjunctivitis,” “Meningococcal,” and “conjunctivitis.” This outbreak occurred in a group of young persons with few comorbidities, all of whom had recently received the quadrivalent meningococcal vaccine, which is not expected to protect against the unencapsulated organisms detected in this outbreak. Whole genome sequencing was used to determine that the outbreak was caused by an unencapsulated strain, supporting the decision to treat with topical antibiotics only, which was associated with rapid improvement of patient symptoms. The source of this outbreak and mechanism of transmission was not identified.

Bacterial conjunctivitis in adults involves direct inoculation into conjunctival membranes without entering the bloodstream or central nervous system. The estimated *N. meningitis* nasopharyngeal carriage rate (including encapsulated and unencapsulated strains) is as high as 5%–10% in some U.S. populations, with a peak prevalence of 23.7% in adults aged 19 years (*6*). Because the average age of a military trainee includes this age, colonization with *N. meningitidis* is expected to be high. Despite this high likelihood of colonization, invasive meningococcal disease is rare, and only one case of meningococcal meningitis has been previously reported among military trainees at this base in 2021 (*7*,*8*). Characterization of the isolates early in the outbreak was critical to understanding the risk for invasive meningococcal disease.

Previous studies, primarily among children before meningococcal vaccines were widely available, led some countries to recommend systemic therapy for patients with meningococcal conjunctivitis; however, no such recommendation exists in the United States (*9*). In this immunocompetent population with recent quadrivalent meningococcal vaccination, cases of conjunctivitis were caused by an unencapsulated pathogen, and no cases of systemic invasive disease occurred. With the exception of one patient who received intravenous antibiotics for periorbital cellulitis, patients with meningococcal conjunctivitis in this setting rapidly improved with topical moxifloxacin, ciprofloxacin, or erythromycin only. If characterization of isolates is not available, systemic therapy might be warranted.

Given the timing of the *N. meningitidis* cases and the association with trainees who had received penicillin (but not azithromycin) streptococcal prophylaxis, the outbreak might be related to the *penA* gene mutation conferring decreased sensitivity to penicillin noted in isolates from the first two cases, combined with the subsequent decline in serum levels of benzathine penicillin approximately 3 weeks after administration (*10*). A definitive cause of the outbreak was not identified during this period.

### Limitations

The findings in this report are subject to at least two limitations. First, only the first two isolates underwent whole genome sequencing. Because meningococcal conjunctivitis is a rare disease, cases in the outbreak were treated as if all the isolates were clonal. However, with unknown carriage of *N. meningitidis* in this cohort, other isolates were possibly introduced during the several months of the public health response. In addition, the original isolate might have mutated during the outbreak, resulting in a change in antimicrobial resistance or virulence; however, cases identified throughout the outbreak responded to topical antimicrobial treatment. Second, because this outbreak occurred in a group of healthy, young military trainees with few underlying medical comorbidities, the findings might not be generalizable to other settings or populations. 

### Implications for Public Health Practice

This report demonstrates the role for serogrouping noninvasive *N. meningitidis* isolates during a possible outbreak to help guide the public health response. In this outbreak in a healthy population, meningococcal conjunctivitis caused by an unencapsulated strain was adequately treated with topical antibiotics and did not lead to severe ocular involvement or systemic infections. Conjunctivitis caused by nongroupable *N. meningitidis* might not require systemic treatment in immunocompetent persons if cases are promptly identified (by group) and appropriately treated, even among persons at high risk for person-to-person exposure living in congregate settings.

## Footnotes

* Reference number FWH20250060N.

## References

[1] Bernstein SH, Feldman HA, Harper OF Jr, Klingensmith WH. Massoral penicillin prophylaxis in control of streptococcal disease. AMA Arch Intern Med 1954;93:894–8. 10.1001/archinte.1954.00240300088008 13157675

[2] Azari AA, Arabi A. Conjunctivitis: a systematic review. J Ophthalmic Vis Res 2020;15:372–95. 10.18502/jovr.v15i3.7456 32864068 PMC7431717

[3] Joussen AM, Funke G, Joussen F, Herbertz G. *Corynebacterium macginleyi*: a conjunctiva specific pathogen. Br J Ophthalmol 2000;84:1420–2. 10.1136/bjo.84.12.1420 11090486 PMC1723327

[4] Rubis A, Schillie S. Meningococcal disease. Manual for the surveillance of vaccine preventable diseases. 5th ed. https://www.cdc.gov/surv-manual/php/table-of-contents/chapter-8-meningococcal-disease.html. Accessed October 30, 2024.

[5] Clark SA, Heymer E, Campbell H, Clinical and microbiological characteristics of meningococcal eye infections: retrospective national surveillance in England, 2010-2022. Clin Infect Dis 2025;ciaf274; Online ahead of print. 10.1093/cid/ciaf274 40452554

[6] Christensen H, May M, Bowen L, Hickman M, Trotter CL. Meningococcal carriage by age: a systematic review and meta-analysis. Lancet Infect Dis 2010;10:853–61. 10.1016/S1473-3099(10)70251-6 21075057

[7] Marcus JE, Bennett WN, Frankel DN, Response to a serogroup B meningococcal disease case among military trainees. Open Forum Infect Dis 2022;9 (5):ofac162. 10.1016/j.ypmed.2018.10.023 35493127 PMC9043002

[8] Broderick MP, Phillips C, Faix D. Meningococcal disease in US military personnel before and after adoption of conjugate vaccine. Emerg Infect Dis 2015;21:377–9. 10.3201/eid2102.141037 25625525 PMC4313647

[9] Parikh SR, Campbell H, Mandal S, Ramsay ME, Ladhani SN. Primary meningococcal conjunctivitis: summary of evidence for the clinical and public health management of cases and close contacts. J Infect 2019;79:490–4. 10.1016/j.jinf.2019.10.015 31669376

[10] Neely M, Kaplan EL, Blumer JL, Faix DJ, Broderick MP. A population pharmacokinetic modeling approach shows that serum penicillin G concentrations are below inhibitory concentrations by two weeks after benzathine penicillin G injection in the majority of young adults. Antimicrob Agents Chemother 2014;58:6735–41. 10.1128/AAC.02744-14 25182635 PMC4249386

